# Sermon by the Lord Bishop of London at St. Thomas's Hospital

**Published:** 1886-12-11

**Authors:** 


### The Guild of St. Luke.

Sermon by the Lord Bishop of Lincoln, at St. Thomas's Hospital.

A service was held on Tuesday last in connection with this Guild, in the Chapel of St. Thomas's Hospital. There was a large congregation. The prayers were said by the Rev. J. Grant Mills, Hospitaller of St. Thomas's, and the Rev. J. F. Underwood, Subwarden of the Guild of St. Luke. The first lesson was read by the Rev. G. Greenwood, Warden of the Guild, and formerly Hospitaller of St. Thomas's, and the second lesson by the Rev. J. W. Hicks, M.A., M.D., Fellow and Dean of Sidney Sussex College, Cambridge, formerly a student of St. Thomas's Hospital. Amongst the other clergy present were the chaplains of Guy's, St. Mary's, and Charing Cross Hospitals; the Rev. E. Walpole Warren, Vicar of Holy Trinity, Lambeth; the Rev. G. H. A. Bromfield, Rural Dean of Lambeth, and Vicar of St. Mary the Less, and the Rev. C. T. Greene, Rural Dean and Rector of Clapham. The offertory was given to the Guild Medical Missionary Fund.

The sermon was preached by the Right Reverend the Lord Bishop of Lincoln, the text being taken from the second Psalm. In the course of his sermon, which was simple, but highly philosophical, the Bishop said that he desired to present for sober consideration *six words*, each of them the seed and centre of important practical ideas. The first word was "Duty." Practically every man admitted the *idea* of duty, of right and wrong. He allowed that certain things "ought" to be done, and certain other things "ought not" to be done. But whence came the idea of "Duty"? The answer to this question brought him to his second word, which was "Conscience." Man had a "conscience." Whether this was a developed faculty or a natural attribute,—something inherent in man as man—he did not stop to inquire. There was the fact—man had a "conscience," and his conscience impelled him to recognise duty. But it did more, it pointed to a great mystery outside himself: to the idea of a being to whom duty was owed, and so he came to his third word—"God." Conscience regarded duty, and pointed to "God," to whom duty was owed. But God could not be understood by the unaided reason. The world had never by its own searching found out God. The great nations of antiquity, and the speculative Eastern peoples of the modern times, had alike failed. The idea of God necessitated a further idea, and that was expressed by his fourth word—"Revelation." God had given a revelation, had made Himself known to men by words and institutions; but above all He had made Himself known by a person—by "Jesus Christ"; and that was his fifth word—"Christ"—the manifestation of God to men. He counselled them to consider what Christ, and the acceptance of Christ, meant to each one of them. Lastly, his sixth word was "The Church": the Church founded and preserved by Christ for the teaching of men and for the giving of the sacraments. He urged that in the hurry of busy life each one should still regard himself as having a special and personal relation to these ideas, and above all to God. Every man must in a sense live his life "alone." He must separate himself from his fellows and determine for himself where he should stand, what position he should take up on these great and fundamental questions. Should he take the positive view! Should he admit that these things were philosophically true! Then reason demanded that a further step be taken, and that these ideas be made actual in each one's life.

The Bishop preached from notes, and, as this brief sketch is entirely from memory, an apology is offered beforehand for any unintentional misrepresentations it it may contain. Among those present were the Hon. Reginald Capel, Mr. Alderman Stone, Treasurer of the Hospital, Mr. George Cowell, Mr. John Croft, Mr. A. O. Mackellar, Mr. B. Pitts, Dr. Mackenzie, Mr. Rendle, Mr. Denison, Mr. Battle, Mr. Ritchie, and a very large number of students. The choir consisted of the sisters and nurses of the hospital.

